# Real-life outcomes of subthreshold laser therapy for diabetic macular edema

**DOI:** 10.1186/s40942-020-00268-3

**Published:** 2021-01-09

**Authors:** Renato M. Passos, Fernando K. Malerbi, Marindia Rocha, Maurício Maia, Michel E. Farah

**Affiliations:** 1 Federal University of Sao Paulo (UNIFESP/EPM), Sao Paulo, SP Brazil; 2 Instituto da Visão (IPEPO), Rua Borges Lagoa 1083, São Paulo, SP 04038-032 Brazil

**Keywords:** Diabetic retinopathy, Diabetic macular edema, Subthreshold micropulse laser, Non-damaging retinal laser, Retinal photocoagulation

## Abstract

**Background:**

Diabetic macular edema (DME) is a major cause of visual impairment and its treatment is a public health challenge. Even though anti-angiogenic drugs are the gold-standard treatment, they are not ideal and subthreshold laser (SL) remains a viable and promising therapy in selected cases. The aim of this study was to evaluate its efficacy in a real-life setting.

**Methods:**

Retrospective case series of 56 eyes of 36 patients with center-involving DME treated with SL monotherapy. Treatment was performed in a single session with the EasyRet® photocoagulator with the following parameters: 5% duty cycle, 200-ms pulse duration, 160-µm spot size and 50% power of the barely visible threshold. A high-density pattern was then applied to the whole edematous area, using multispot mode. Best corrected visual acuity (BCVA) and optical coherence tomography (OCT) data were obtained at baseline and around 3 months after treatment.

**Results:**

Fifty-six eyes of 36 patients were included (39% women, mean age 64.8 years old); mean time between treatment day and follow-up visit was 14 ± 6 weeks. BCVA (Snellen converted to logMAR) was 0.59 ± 0.32 and 0.43 ± 0.25 at baseline and follow-up, respectively (p = 0.002). Thirty-two percent had prior panretinal photocoagulation (p = 0.011). Mean laser power was 555 ± 150 mW and number of spots was 1,109 ± 580. Intraretinal and subretinal fluid (SRF) was seen in 96 and 41% of eyes at baseline and improved in 35 and 74% of those after treatment, respectively. Quantitative analysis of central macular thickness (CMT) change was performed in a subset of 23 eyes, 43% of which exhibited > 10% CMT reduction post-treatment.

**Conclusions:**

Subthreshold laser therapy is known to have RPE function as its main target, modulating the activation of heat-shock proteins and normalizing cytokine expression. In the present study, the DME cases associated with SRF had the best anatomical response, while intraretinal edema responded poorly to laser monotherapy. BCVA and macular thickness exhibited a mild response, suggesting the need for combined treatment in most patients. Given the effect on SRF reabsorption, subthreshold laser therapy could be a viable treatment option in selected cases.

## Background

Diabetic macular edema (DME) remains the leading cause of visual impairment in patients with diabetes mellitus, while also being one of the leading causes of legal blindness worldwide [1]. Recent studies by the Diabetic Retinopathy Clinical Research Network (DRCR.Net) with level A of evidence have demonstrated that the gold standard treatment for DME is the combination of ranibizumab with deferred laser photocoagulation, which proves to be superior to laser monotherapy or triamcinolone in terms of visual acuity gain and anatomical improvement [2]. Other studies with anti-angiogenic drugs and corticosteroids have also shown good results, both in anatomical and functional outcomes [3–5]. However, disadvantages are the short duration of intravitreal drugs, the need for repeated injections, frequent visits and ancillary examinations, the safety issues (risk of endophthalmitis, intraocular pressure increase and cataracts in case of corticosteroids), and the high economic burden of DME treatment for patients and health systems worldwide [6], and all factors combined make the search for lower cost and safer treatment modalities an absolute priority.

The Early Treatment Diabetic Retinopathy Study (ETDRS) [7] had already demonstrated the beneficial effect of laser photocoagulation for treating clinically significant DME, reducing the chance of visual loss by 50% in 3 years despite its adverse effects such as retinal scarring, permanent scotomata, among others. Conversely, recent understanding of the modification of gene expression mediated by the healing response of the RPE to thermal injury [8] suggests that the useful therapeutic cellular cascade is activated not by laser-killed RPE cells, but by the still-viable RPE cells surrounding the burned areas that are reached by the heat diffusion at sublethal thermal elevation [9]. In the years that followed, other authors evaluated different non-damaging macular laser modalities and strategies for DME treatment, with satisfactory results that were usually superior to that with conventional macular laser as proposed by ETDRS [9–13]. Among these, subthreshold micropulse laser stands out as a safer, non-scarring procedure, causing no tissue damage evidenced by imaging modalities and microperimetry analyses [14–18]. Besides the usual 810-nm wavelength already proven effective [9, 19, 20], yellow (577-nm) wavelength has also shown good success and safety [21, 22], due to its intrinsic physiobiological characteristics, namely better penetration through media opacities, null absorbance by macular xanthophyll pigments and an excellent combined absorbance by melanin and oxyhemoglobin [23].

Anatomic measures on spectral-domain optical coherence tomography (OCT), such as precise evaluation of individual layers, quantification of retinal thickness and macular volume, qualitative assessment of fluid distribution and other so-called OCT biomarkers, could predict treatment success or failure with various therapies for DME, such as intravitreal anti-VEGF or corticosteroids [24]. However, such analysis has not been done to predict tissue response to subthreshold laser therapies. Therefore, this study aimed to evaluate the clinical and anatomical response of subthreshold micropulse laser as monotherapy for DME patients in a real-life setting, with a short follow-up.

## Methods

This was a retrospective, single-center, case series that analyzed patients with a diagnosis of type I or II diabetes mellitus and center-involving macular edema seen at *Instituto da Visão* (IPEPO), São Paulo-SP, Brazil, from July 2018 to September 2019. The study was conducted according to the Declaration of Helsinki and approved by the Federal University of São Paulo (UNIFESP) research ethics board.

In that timeframe, anti-angiogenic treatment was unavailable due to issues with the funding public agency. Therefore, all patients who presented with center-involving DME with visual acuity (VA) worse or equal to 20/40 were offered subthreshold laser (SL) monotherapy in one eye or both in case both were involved. Since the purpose of the study was to reflect a real-life setting with heterogeneous disease severities, the inclusion criteria were more flexible than usual: (a) diagnosis of type I or II diabetes mellitus; (b) minimum age of 18 years-old; (c) center-involving DME with no minimum or maximum OCT macular thickness; (d) VA worse or equal to 20/40; (e) availability of baseline and follow-up OCT images. The exclusion criteria were (a) presence of high-risk proliferative retinopathy and (b) concomitant ocular conditions that might impair treatment response or the analysis of the results such as advanced glaucoma, uveitis, dense cataracts, age-related macular degeneration, or other maculopathies. The time of DME diagnosis, history of previous treatments older than 6 months and systemic blood glucose control were not considered as exclusion criteria.

Baseline examination included: best corrected visual acuity (BCVA) measured by Snellen chart, slit-lamp examination, fundus biomicroscopy, fluorescein angiography and OCT. After the initial workup, patients underwent SL treatment and were asked to return in about 3 months post-treatment, with a follow-up OCT and clinical evaluation. This approximate timeframe was chosen to allow for the laser to perform its action, since it is known to take a longer time than usually seen with intravitreal drugs. Despite being a relatively short follow-up, the objective was to verify a short-term tissue response to the SL treatment. It is true that laser effects may persist for longer periods; however, it is unusual that an initial non-responder patient will exhibit any significant response in the following months unless offered retreatment or another rescue therapy. Other relevant studies on the topic such as Lavinsky et al. [9], Luttrull et al. [19] and Vujosevic et al. [21] had already demonstrated significant effects on macular thickness and visual acuity as soon as 3 months following a single session of SL application.

Other variables gathered from the patients’ medical charts included: sex, age, eye laterality, assisting doctor (RMP or FKM), OCT and laser treatment dates, the timeframe between said dates (in weeks), lens status, diabetic retinopathy severity grading, previous panretinal photocoagulation (PRP) or macular laser, SL power and number of laser spots per treatment session.

### OCT quantitative and qualitative analyses

Since we performed a retrospective analysis, we found out when collecting the data that some patients had their baseline and follow-up OCTs performed with different instruments (HRA Spectralis® by Heidelberg, Heidelberg, Germany; DRI-OCT Triton® by Topcon, Oakland NJ, USA or Avanti® by Optovue, Fremont CA, USA), which could make some comparisons unreliable such as central macular thickness (CMT). In those patients with baseline and follow-up OCTs with the same instrument and reliable follow-up scans, CMT obtained by the central 1-mm ETDRS map was measured. The following qualitative biomarkers were also analyzed: presence or absence of intraretinal fluid (IRF) and subretinal fluid (SRF) at baseline, worsening, maintenance or improvement of those fluid types at follow-up and integrity of ellipsoid zone at baseline. These parameters were evaluated even in the case of different OCT devices, due to their qualitative nature, since excluding these data would diminish the strength of the analysis.

### Laser treatment technique

After mydriasis with tropicamide 1% and phenylephrine 10% and topical anesthesia with proxymetacaine eye drops, an Area Centralis (Volk Opticals, Mentor, OH, USA) contact lens was applied. The EasyRet® machine, which features a pure 577-nm wavelength laser cavity (Quantel Medical, France) was used to perform the SL treatments. Following the manufacturer’s guideline for subthreshold macular treatment, the laser was set to Subliminal® mode with the following parameters: spot size of 160 µm selected on the laser (equivalent to 150 µm at the retina), duty cycle of 5% and pulse duration of 0.2 s. Power was titrated to set the thermal threshold for each patient, by increasing the power level until a barely visible burn was obtained in an area of non-edematous retina in the perimacular region. Power was then reduced by 50%, and the treatment was performed in all edematous areas using the Multispot mode with zero spacing between spots, in a high-density fashion [9].

### Statistical analysis

Descriptive and statistical analyses were performed using IBM SPSS Statistics for Windows, version 23.0 (IBM Corporation, Armonk, NY, USA). Patients’ characteristics and quantitative variables are presented in terms of mean and standard deviation (SD). A paired 2-tailed Student *t*-test was used for continuous variables, and the chi-square test was used to compare proportions for categorical variables whenever possible; Fisher’s exact test was applied in cases of expected counts less than 5. Statistical significance was set at *p* < 0.05.

## Results

A total of 56 eyes of 36 patients with complete data were analyzed. These patients included 14 women (38.8%) and 22 men (61.1%), with a mean age of 64.8 ± 7.8 years (range 47–77 years). There were 18 eyes previously treated with PRP, all performed more than 6 months before enrollment in the study. The mean duration between treatment date and follow-up visit was 14.1 ± 6.3 weeks. All treatment sessions were performed by a retinal surgeon (RMP or FKM) with expertise in diabetic retinopathy treatment. Table 1 summarizes the baseline characteristics and treatment parameters.

**Table 1:** Summary of baseline characteristics and treatment parameters of patients with diabetic macular edema treated with subthreshold micropulse laser monotherapy

Age (years), mean ± SD	64.8 ± 7.8
Women %	38.8
Pseudophakic %	17.8
NPDR %	44.6
Previous panphotocoagulation %	32.1
Previous macular laser %	21.4
Intraretinal fluid %	96.4
Subretinal fluid %	41.1
Ellipsoid disruption %	57.1
Follow-up (weeks), mean ± SD	14.1 ± 6.3
BCVA logMAR, mean ± SD	0.59 ± 0.32
Laser power (mW) mean ± SD	555 ± 150.7
Number of spots (n), mean ± SD	1,109.7 ± 580.4
Retreatment %	17.8

**Fig. 1:**
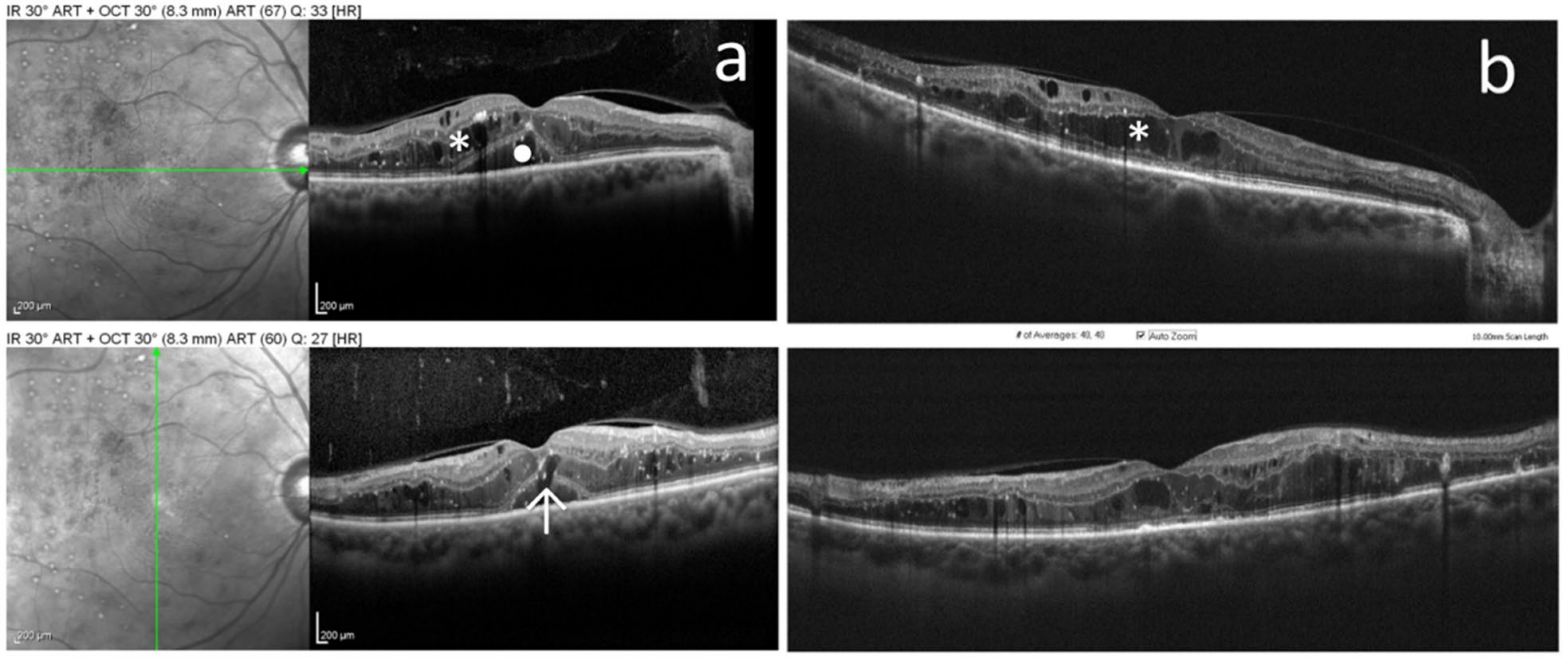
**a** Baseline OCT depicting intraretinal fluid (*) and subretinal fluid (•), plus a focal disruption of ellipsoid zone (arrow) in the subfoveal area. **b** 3 months after SL treatment showing resolution of SRF but no visible effect on IRF (VA remained 20/60)

Baseline BCVA was 0.59 ± 0.32 logMAR, which improved to 0.43 ± 0.25 logMAR (p = 0.002) at follow-up. Qualitative analysis revealed that intraretinal fluid (IRF) was present in 54 (96.4%) eyes at baseline; at the follow-up visit, 19 (35%) eyes showed resolution or improvement of this parameter. Subretinal fluid (SRF) was present in 23 (41.1%) eyes at baseline; at the follow-up visit, 17 (74%) eyes showed resolution or improvement of this parameter. IRF improvement was associated with VA gain (p = 0.018) while SRF resolution or improvement was not (p = 0.343). Disruption of the ellipsoid zone at baseline OCT was present in 32 (57.1%) eyes. The presence of this biomarker was not associated with VA change at follow-up (p = 0.779). The presence of previous PRP was associated with a better functional outcome (p = 0.011). Figure 1 depicts a nice illustrative case.

**Fig. 2:**
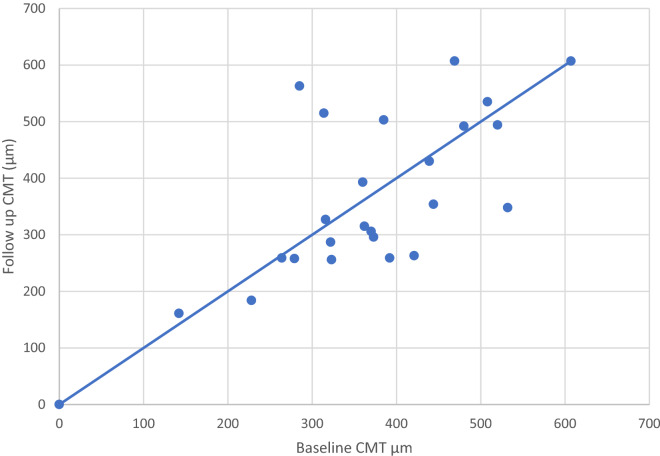
Histogram depicting baseline (x axis) and follow up (y axis) central macular thickness values (µm) for 23 eyes evaluated in the same OCT device. All dots below the reference line represent eyes that had CMT reduction at follow up with laser monotherapy

Absolute quantitative analysis of CMT change could not be performed throughout the whole sample as only 23 eyes had both baseline and follow up CMT measurements performed with the same OCT device. Due to the reduced number of patients per OCT device, statistical analysis was not carried out for quantitative parameters. Regarding only those eyes that had baseline and follow up evaluation with the same device, fifteen (65%) had a reduced CMT at follow-up (p = 0.815), and 10 (43%) eyes showed a CMT reduction > 10% of baseline CMT (Table 2; Fig. 2).

**Table 2:** Central macular thickness (CMT) obtained from 23 eyes that underwent baseline and follow up evaluations on the same OCT device, which allowed a quantitative analysis

Eye	Baseline CMT (µm)	Follow up CMT (µm)	CMT change (µm)	Thickness reduction %
1^a^	444	354	− 90	*20.27*
2^a^	316	327	11	− 3.48
3^a^	370	306	− 64	*17.30*
4^a^	421	263	− 158	*37.53*
5^a^	385	503	118	− 30.65
6^a^	264	259	− 5	1.89
7^a^	142	161	19	− 13.38
8^a^	360	393	33	− 9.17
9^a^	480	492	12	− 2.50
10^a^	322	287	− 35	*10.87*
11^a^	228	184	− 44	*19.30*
12^b^	532	348	− 184	*34.59*
13^b^	362	315	− 47	*12.98*
14^b^	520	494	− 26	5.00
15^b^	508	535	27	− 5.31
16^b^	279	258	− 21	7.53
17^b^	373	296	− 77	*20.64*
18^b^	285	563	278	− 97.54
19^b^	323	256	− 67	*20.74*
20^b^	314	515	201	− 64.01
21^b^	392	259	− 133	*33.93*
22^b^	439	430	− 9	2.05
23^b^	469	607	138	− 29.42
Mean ± SD	370.8 ± 98.4	365.4 ± 127	− 5.4	1.46

Italic values indicate eyes that had CMT reduction greater than 10% of basal CMT with laser monotherapy. The mean reduction in CMT was not statistically significant (p = 0.815)

^a^ Baseline and follow up OCT exams performed on the Avanti RTVue device

^b^ Baseline and follow up OCT exams performed on the Triton Topcon device

In a sub analysis, we investigated only phakic eyes with a clear lens or pseudophakic eyes without posterior capsule opacification. It did not yield different associations regarding baseline retinal biomarkers and VA change at follow-up. No complications related to the treatment were encountered in this series, even though no tests were performed to specifically investigate it (autofluorescence/microperimetry). None of the patients underwent any other treatment for DME, including anti-VEGF injections, during the follow-up period. Some patients were retreated with a second SL application after 4 months at the surgeon’s discretion (17.8% of eyes).

## Discussion

The present study describes a case series of patients treated with subthreshold micropulse laser as monotherapy, in a real-life setting, which meant various degrees of DME severity and chronicity and of visual impairment. The reality in many centers relying on governmental funding is that they do not have the best treatment modalities available for the population that depends on those health instances. Having access to alternative treatments seems to be of utmost importance in such settings.

As previously mentioned, there are several inconveniences of continuous or frequent intravitreal treatments for DME. DRCR.Net [2], RISE/RIDE [3], VIVID/VISTA [4] and other studies have discussed the importance of repeated intravitreal injections of anti-VEGF drugs to maintain the initial visual and anatomical gains. Even though the number of injections decrease in the following years (in many cases because of flexibilization of *pro re nata* regimens, patient dropout and other non-medical reasons), the recurrence of macular edema and fluctuation in visual acuity are expected. For that reason, the concepts of rescue treatments, treatment switching and treatment combinations are always extremely relevant.

Macular laser has always been present in one way or another in those trials, frequently used as rescue treatment in case of persistent or recurrent edema. However, the way it has been usually done, using photocoagulation parameters established in the 1980s and with the negative adverse effects of tissue scarring, paracentral scotomata and reduced macular sensitivity [7], the benefits of laser may have been overshadowed or even absent. Several modalities of tissue sparring, subthreshold macular lasers or photostimulation treatment strategies have been described and thoroughly investigated in the last 20 years [10, 11], but their discussion in depth is beyond the scope of this article.

The treatment used in this study was a 577-nm subthreshold micropulse laser (Subliminal® brand by Quantel Medical, France) using a 5% duty cycle and the parameters already described. The intention of using it as monotherapy and an alternative to anti-VEGF was to evaluate the patterns of clinical response in the short term (average 14 weeks), both functional (BCVA) and anatomical (OCT). As these lasers are known to exert their main physiobiological effect on the RPE cells due to their absorption profile by melanin and choriocapillaris oxyhemoglobin [23], we were not surprised to confirm its higher efficacy in SRF absorption (74% of cases that presented with SRF at baseline improved at follow-up) and not so much regarding IRF (only 35% of cases improved, but those were associated with better VA, p = 0.018). The evaluation of OCT biomarkers have been increasingly valued for several macular diseases [24], yet most studies on subthreshold micropulse lasers for DME focus on the CMT or macular volume as an anatomical outcome with no specific attention to fluid distribution. CMT could not be properly analyzed in the present study because different OCT instruments were utilized in the follow-up of most patients; only 23 eyes of 18 patients had the baseline and follow-up OCT done with the same instrument. Among those, 65% showed a decrease in CMT after treatment, with 43% showing a larger than 10% decrease (Table 2; Fig. 2).

Regarding VA, the mean difference of − 0.16 logMAR post treatment was statistically significant (p = 0.002). However, there was no association between VA gain and baseline ellipsoid disruption or SRF improvement (only IRF improvement). Prior PRP has been associated with VA gain (p = 0.011). This was discussed extensively in our previous study that showed an improvement of macular edema 12 months after PRP in around 50% of patients [25]. We believe that the higher oxygen tension in the vitreous cavity and retina [26] and lower VEGF expression in previously panphotocoagulated eyes seem to facilitate DME control, which is consistent with the data obtained in the present study.

Subthreshold micropulse laser parameters and titration protocols vary significantly between studies and have always been subject to intense criticism in the literature. In our study, the mean laser power was 555 ± 150 mW (range 350–1150) and the mean number of spots 1109 ± 580 (range 234–2474), reflecting a high variability in the sample. It must be reinforced that there is no strong recommendation so far about ideal parameters for DME treatment, since comparative prospective studies are scarce. Some authors advocate fixed parameters while others defend varied methods of titration [10].

One factor that may have contributed to a sub-par therapeutic performance of SL monotherapy in the present case series was the disease severity and chronicity of most cases, which might be less of an issue in randomized clinical trials with strict inclusion and exclusion criteria, and milder DME cases. Most patients seen at our institution have a very low socioeconomic status and poor metabolic control, and few have access to an endocrinologist [27]. Many cases of DME had baseline OCTs exhibiting intraretinal hard exudates, confluent degenerative cystic edema and disrupted ellipsoid zones (57.1% of cases), suggesting chronically ill retinas that might not respond ideally to the laser’s regenerative stimulus.

It seems that severe structural changes in the neurosensory retina and microvasculature seen at late stages of DME may prevent a good response with subthreshold laser alone, which relies on the existence of a viable intracellular machinery to work properly and a certain degree of cell viability and retinal integrity [10]. While SL produces clinical effects akin to pharmacological therapy, the slower onset and lasting effect suggest that these changes, like conventional photocoagulation, are mediated by secondary laser-induced modulation in RPE cellular function [28]. Progressive improvement in macular edema has been observed for several years after a single session of subthreshold macular laser treatment, suggesting a long-lasting effect [10]. This might point to a potential benefit of combination therapy with anti-VEGF agents, relying on a theoretical synergistic effect. There would be a quick and effective early response from anti-VEGF leading to a decrease in macular thickness, followed by SL applied over a thinner macula and its long-lasting protective effect provided by RPE remodeling and improved cytokine expression [29]. This is currently being investigated by our group in collaboration with the Pan-American Collaborative Retina Study Group (PACORES) in a multicenter prospective clinical trial.

Limitations of the present study included: retrospective design, different OCT instruments precluding an adequate objective analysis, BCVA information not collected in a standardized manner (i.e., ETDRS letters), short follow-up in some cases and too long in others, missing clinical data regarding glycemic and blood pressure control, diabetes duration and diabetes treatment regimen. Strengths of the study included: real-life evidence, description of a potential alternative treatment to anti-VEGF in services where this may be relevant and a greater emphasis on interpretation of OCT biomarkers as predictors of functional and anatomical response to subthreshold macular laser.

In conclusion, we present our experience with SL therapy in an average number of patients as monotherapy for DME and an alternative to anti-VEGF injections. This may be relevant especially from a cost-effective standpoint or in services that cannot provide patients with the standard-of-care treatments such as in our case. We also discuss the possibility of utilizing SL in a combination therapy to reduce the burden of repeated injections for an adequate anatomic control and visual stability in DME patients.

## Data Availability

Not applicable.

## Abbreviations

BCVA: Best corrected visual acuity
CMT: Central macular thickness
DME: Diabetic macular edema
ETDRS: Early treatment diabetic retinopathy study
IRF: Intraretinal fluid
logMAR: logarithm of the minimum angle of resolution
NPDR: Non-proliferative diabetic retinopathy
OCT: Optical coherence tomography
PRP: Panretinal photocoagulation
RPE: Retinal pigment epithelium
SRF: Subretinal fluid
SL: Subthreshold laser
VA: Visual acuity
VEGF: Vascular endothelial growth factor
